# Are acoustics enough? Semantic effects on auditory salience in natural scenes

**DOI:** 10.3389/fpsyg.2023.1276237

**Published:** 2023-11-30

**Authors:** Sandeep Reddy Kothinti, Mounya Elhilali

**Affiliations:** Department of Electrical and Computer Engineering, Center for Language and Speech Processing, The Johns Hopkins University, Baltimore, MD, United States

**Keywords:** auditory salience, auditory attention, audio event detection, bottom-up attention, auditory perception

## Abstract

Auditory salience is a fundamental property of a sound that allows it to grab a listener's attention regardless of their attentional state or behavioral goals. While previous research has shed light on acoustic factors influencing auditory salience, the semantic dimensions of this phenomenon have remained relatively unexplored owing both to the complexity of measuring salience in audition as well as limited focus on complex natural scenes. In this study, we examine the relationship between acoustic, contextual, and semantic attributes and their impact on the auditory salience of natural audio scenes using a dichotic listening paradigm. The experiments present acoustic scenes in forward and backward directions; the latter allows to diminish semantic effects, providing a counterpoint to the effects observed in forward scenes. The behavioral data collected from a crowd-sourced platform reveal a striking convergence in temporal salience maps for certain sound events, while marked disparities emerge in others. Our main hypothesis posits that differences in the perceptual salience of events are predominantly driven by semantic and contextual cues, particularly evident in those cases displaying substantial disparities between forward and backward presentations. Conversely, events exhibiting a high degree of alignment can largely be attributed to low-level acoustic attributes. To evaluate this hypothesis, we employ analytical techniques that combine rich low-level mappings from acoustic profiles with high-level embeddings extracted from a deep neural network. This integrated approach captures both acoustic and semantic attributes of acoustic scenes along with their temporal trajectories. The results demonstrate that perceptual salience is a careful interplay between low-level and high-level attributes that shapes which moments stand out in a natural soundscape. Furthermore, our findings underscore the important role of longer-term context as a critical component of auditory salience, enabling us to discern and adapt to temporal regularities within an acoustic scene. The experimental and model-based validation of semantic factors of salience paves the way for a complete understanding of auditory salience. Ultimately, the empirical and computational analyses have implications for developing large-scale models for auditory salience and audio analytics.

## 1 Introduction

In its most general definition, attention can be described as an information selection process that facilitates the brain's ability to select the most relevant information in the sensory space while filtering out less relevant information (Posner and Petersen, 1990; Driver, 2001). Defining what is relevant depends on several perceptual and cognitive processes, and the factors governing these processes are often categorized as either top-down or bottom-up factors (Corbetta and Shulman, 2002; Theeuwes, 2010). Top-down or endogenous attention is a voluntary or goal-driven process that aligns with the observer's behavioral goals (Baluch and Itti, 2011). Alternatively, bottom-up or exogenous attention is stimulus-driven, where the sensory signal automatically captures an observer's attention (Theeuwes, 1991; Schreij et al., 2008; Kaya and Elhilali, 2014). In auditory perception, the former mode of attention describes our voluntary selection of a friend's voice in a busy cafeteria while ignoring background chatter. The latter form of attention often manifests in the same setting where our phone ringing will inadvertently grab our attention and that of others around us. This interplay and competing demands on our attentional control are at the core of what enables our brain to make sense of the cacophony of sounds that enter our ears at every moment in time and focus limited processing capacity on the contingencies of that instant.

While studies of top-down auditory attention often invoke a variety of behavioral and cognitive tasks to probe manifestations of top-down control on sensory processing, studying bottom-up auditory attention continues to be a challenge. Studies of bottom-up attention need to examine a listener's attentional state without explicitly directing their attentional control. Organically achieving this balance is often impossible due to the lack of measurable biometric markers to probe a user's engagement with an acoustic stimulus. In contrast, studies of visual perception have leveraged eye tracking (both fixation and eye movement) to quantify the effect of images, scenes, and videos during free-viewing or complex task execution by observers, making it a *de facto* standard measure of bottom-up visual attention (Treue, 2003; Foulsham and Underwood, 2008). Numerous studies have shown that analysis of conspicuous or salient regions in a visual input can account for predictions of eye gaze patterns, and that human observers consciously access such salience computations to guide perception (Borji et al., 2013; Borji, 2018).

By tapping into the natural behavior of eye movement during free viewing, visual salience studies have revealed a complex interplay of factors guiding attention to the most salient regions in a scene (Koch and Ullman, 1985; Itti and Koch, 2001). Early research focused on low-level visual attributes like contrast, orientation, and color through behavioral experiments like pop-out searches and odd-ball tasks. These attributes underpin popular computational models of salience, explaining center-surround processing in the early visual system (Itti et al., 1998; Itti and Koch, 2000; Peters et al., 2005). They effectively predict human gaze patterns in controlled visual settings (Parkhurst et al., 2002); though these mechanisms fail to generalize to accounts of eye fixation with real-world images with complex object layouts (Foulsham and Underwood, 2008). Psychophysical experiments manipulating semantic and object information while retaining low-level visual cues uncovered systematic effects of higher-order factors, including higher-order statistics, objects, and object identity (Einhäuser et al., 2007; Einhauser et al., 2008; Cerf et al., 2008). These studies observed that human fixations tend to cluster around object centers (Foulsham and Kingstone, 2013). Semantic factors, such as preferences for faces and object-context consistency also influence fixations on objects (Hershler and Hochstein, 2005; Stirk and Underwood, 2007; Cerf et al., 2009). These results paint a more encompassing picture, where low-level visual features and higher-order contextual and semantic factors work dovetail to guide the human gaze in natural vision.

Unlike eye movement, auditory perception has no behavioral parallel to infer auditory perception in a non-intrusive manner. Several experimental paradigms, such as odd-ball paradigms, distraction tasks, and dichotic listening experiments, were suggested as proxies for probing the effects of sensory salience on auditory perception. Deviance detection and particularly odd-ball paradigms examined the premise that contrast in low or high-level statistics of a token relative to its surround results in enhanced neural and perceptual representation, ultimately making it stand out (Schröger and Wolff, 1996; Jacobsen et al., 2003; Grimm and Escera, 2012). Still, the use of odd-ball paradigms in behavioral studies of auditory salience does elicit *active* engagement of listeners, hence confining the organic control of their attentional state by sounds themselves in favor of the task or instructions given (Shuai and Elhilali, 2014; Huang and Elhilali, 2020). Alternative approaches employing distraction or dual tasks also successfully probed various dimensions of auditory salience (Dalton and Lavie, 2006; Duangudom and Anderson, 2007). Owing to the complexities of controlling the attentional state in addition to perceptual and cognitive loads of such paradigms, the majority of these approaches employed simplified stimuli such as tones and noise patterns (Elhilali et al., 2009; Duangudom and Anderson, 2013; Petsas et al., 2016) or controlled sequences of natural sound tokens (Duangudom and Anderson, 2013; Tordini et al., 2015). Dichotic listening (Cherry, 1953; Hugdahl, 2009) has been successfully employed in studies of auditory salience on natural scenes, where two competing stimuli are pitted against one another in different ears (Huang and Elhilali, 2017; Kothinti et al., 2020, 2021) and listeners continuously reported their attentional switches. This paradigm offered the advantage of collecting continuous salience profiles over time and minimized the top-down modulation effects achieved by pairing sound scenes randomly across trials and subjects. Kothinti et al. (2021) showed that online crowd-sourcing of the dichotic salience task reliably reproduced salience data on natural sound scenes, paving the way to scale salience data on natural sounds to large samples from a diverse subject pool. In addition, the use of complex everyday scenes broadens the space of sensory stimuli and allows the investigation of multiple factors at play in guiding a listener's attention.

Using natural scenes to gauge auditory salience also paves the way to exploring the effects of contextual and semantic factors, aspects that have so far been lacking in theoretical and experimental accounts of auditory salience. Contextual effects relate to how a scene (and events within it) unfold over time. It is an important element of auditory scene analysis and perception as natural sounds are characterized by their temporal dynamics which can span multiple timescales from few milliseconds (e.g., speech phonemes) to hundreds of milliseconds (e.g,. syllables) or longer (e.g., words, phrases). Such multiscale temporal integration is a key computational principle for auditory function (Norman-Haignere et al., 2022). Importantly, context is a critical factor in auditory salience as knowing when a sound occurs and how it relates to other changes in the scene shapes its perception. For instance, a sudden loud noise might be less startling in a context where loud noises are expected. Representation of context is shaped by local and global statistics of a scene as sounds unfold over time, and directly informs semantic interpretations of the scene (i.e., recognizing what the sound is, when it occurs and possibly where its source is). Controlling these very attributes of an acoustic scene requires designing an experiment that can eliminate or reduce their effects. One way to control contextual and semantic information is by playing the sound in reverse. Time reversal is a technique that was used as early as 1953 by Cherry as a competing signal during dichotic listening (Cherry, 1953). It has been employed in behavioral and physiological studies in human and animals using speech, music and vocalization sounds both as a control stimulus, but also to investigate sensitivity of the auditory system to both meaning as well as acoustic profiles of the signal (Glass and Wollberg, 1983; Droit-Volet et al., 2013; Mushtaq et al., 2019). When listening to time-reversed audio, the context is reversed, affecting long-range temporal processing and increasing the difficulty of object identification, hence curtailing semantic effects. Time reversal has been shown to reduce the linguistic cues of speech signals (Moore-Parks et al., 2010; Gherri and Eimer, 2011) as well as influence the emotional valence and melodic judgment of music signals (Droit-Volet et al., 2013).

The present study employs time-reversed scenes as a counterpoint to natural listening to probe the role of acoustic, contextual, and semantic factors in guiding auditory salience. We hypothesize that a subset of sound events within natural settings likely invoke similar behavioral responses whether played forward or backward, and that such responses are likely explained by low-level acoustic properties of the event. In contrast, other events will likely be less interpretable when played backward, owing to their contextual and semantic attributes. We hypothesize that such events invoke different responses in forward and backward scenes and that accounts of contextual and semantic attributes can explain this difference. From a computational perspective, we can quantify the higher-level attributes of a sound, which extend beyond low-level acoustics, by harnessing the power of deep neural networks. These models are trained to map the signal onto relevant semantic spaces to recognize events, yielding a nonlinear transformation of the audio signal onto meaningful dimensions that facilitate recognition and detection (Heittola et al., 2018; Mesaros et al., 2021). While the exact interpretation of these network embeddings is highly dependent on the architecture, data and learning strategy, these representations are nonetheless valuable proxies for the contextual and semantic information in audio signals which can then be used to accurately tag the scenes.

The present study aims to quantify the role of effects beyond low-level acoustic features in directing salience to natural scenes. The experimental paradigm employs a dichotic listening task that affords continuous accounts of perceptual salience and enables the adoption of a wide range of acoustic profiles and semantic settings using everyday scenes. We first describe the experimental methods using forward and backward dichotic paradigms. The analysis evaluates differences in behavioral responses between these two paradigms and quantifies factors such as low-level, contextual, and semantic attributes of the natural scenes. These effects are then analyzed in a computational framework to quantify their contributions and ability to account for behavioral salience reported by human listeners.

## 2 Materials and methods

### 2.1 Behavioral procedure

#### 2.1.1 Stimuli

**Figure 1:**
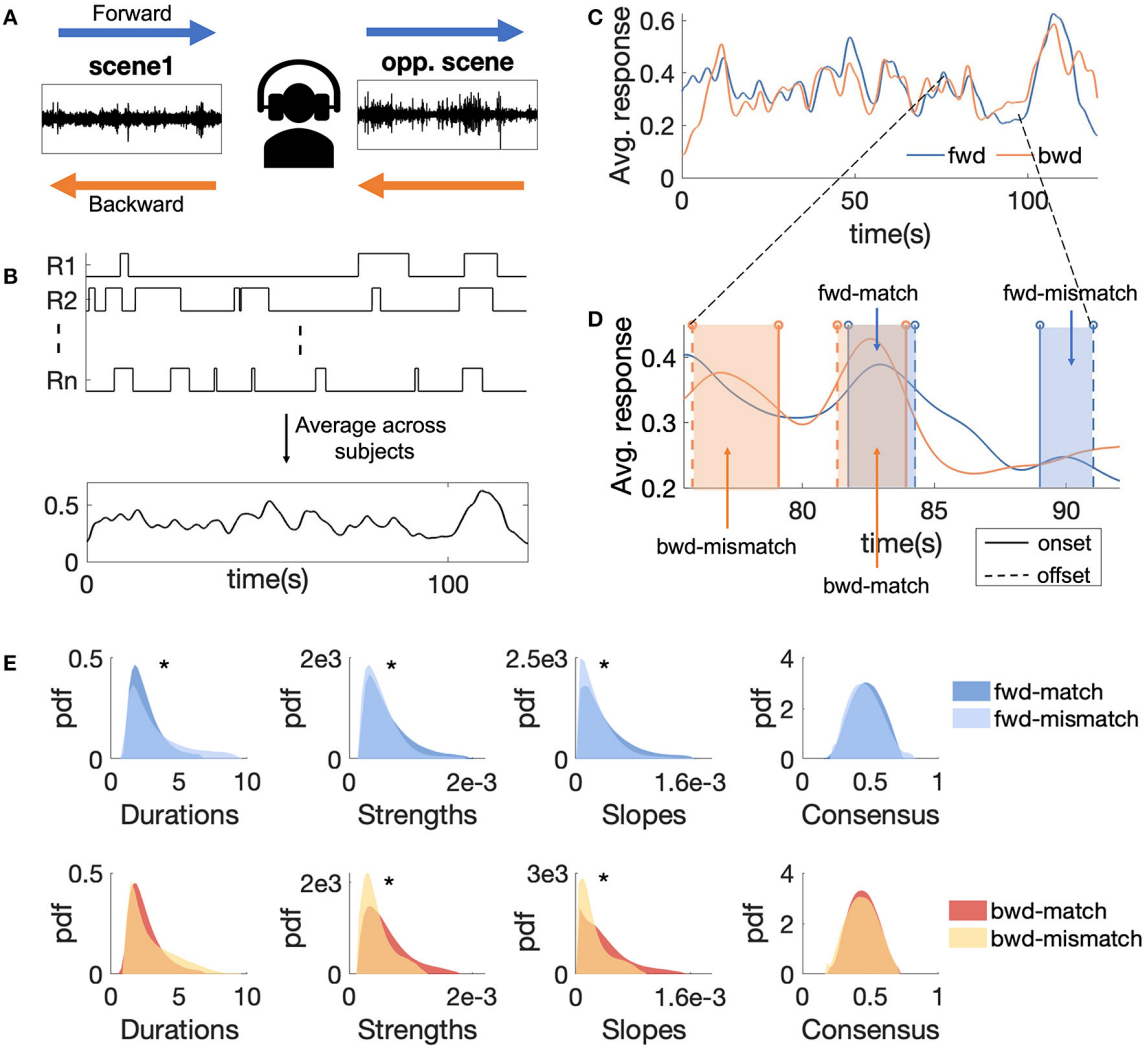
**(A)** Schematic representation of an experimental trial in *fwd* and *bwd* experiments. **(B)** Example of individual subject responses for a single trial and the average behavioral salience for the scene. **(C)** Example average behavioral salience for scene 1 from *fwd* and *bwd*. The response for the *bwd* scene was reversed in time. **(D)** Representative examples of match and mismatch events in *fwd* and *bwd* experiments. Blue rectangles show *fwd* events and orange rectangles show *bwd* events. **(E)** Distributions for matched and mismatched events for (i) duration (ii) strength (iii) slope, and (iv) absolute consensus. The distributions were plotted by using kernel density using Gaussian kernels. Significant differences in medians are indicated by *.

This study used 20 natural audio scenes, × 2 min each (total duration 40 min), as stimuli. These scenes were downloaded from freesound.org (Font et al., 2013) and constituted a range of settings, including speech, music, animals, vehicles, and abstract sources. The sounds were chosen to balance several criteria: scene complexity (few vs. many sources), recording environment (indoor vs. outdoor), and coverage of sound classes. Two separate experiments were conducted on the selected stimuli: A *forward* experiment (referred to hereafter as *fwd*), where the scenes were presented in their original format, and a *backward* experiment (referred to hereafter as *bwd*), where the scenes were time-reversed (Figure 1A). Table 1 shows the details of the scenes, with a short description of audio events present in each one. All scenes were sampled or resampled to 22 kHz and presented with 16-bit encoding per sample. Scenes were normalized with root mean squared (RMS) energy of the loudest 1% of the scene to provide a similar dynamic range for acoustically rich and sparse scenes (Huang and Elhilali, 2017).

**Table 1:** Duration and description of the stimuli used in the study.

**Scene #**	**Duration**	**Description**
1	2:02	Volleyball court, claps, and cheers
2	2:04	Beach, seagulls, kids playing
3	1:56	Birds singing
4	1:59	Street fair, music, cheers
5	2:04	Outdoor party, people talking
6	2:01	Dishes, cutlery
7	2:07	Market, people talking, vehicle horns
8	2:04	Train station, music, announcements
9	1:54	Street music, guitar
10	1:57	Restaurant, people chattering
11	1:56	Music concert, claps, cheers
12	2:03	Motor boat, birds
13	1:56	Restaurant, dishes, people talking
14	2:03	Ocean waves, birds
15	2:02	Machinery, mixing, and rotating sounds
16	2:02	Busy street, vehicle sounds, crowd talking
17	1:55	Top sounds on a table
18	1:55	Inside a bus, announcements
19	2:00	Fire pit, metal clanks, fire crackle
20	1:54	Airplane sounds, birds, squeaky toy

#### 2.1.2 Participants

A total of 268 participants were recruited for this study from the Amazon Mechanical Turk (MTurk) platform for a web-based experiment. Among this pool, 132 listeners participated in the *fwd* experiment, and the remaining 136 participated in the *bwd* experiment. The median age of participants was 29 years [standard deviation (std) = 8 years], with 170 male, 83 female, and 15 non-binary or unspecified gender. While 116 participants reported having no musical experience, 76 participants reported having at least 2 years of musical training. Participants were compensated for taking part in the study after completion. All experimental procedures were approved by the Johns Hopkins University Institutional Review Board (IRB).

#### 2.1.3 Experimental setup

The dichotic listening paradigm, used in earlier auditory salience studies (Huang and Elhilali, 2017; Kothinti et al., 2021), was adopted for experiments in this study. Each experiment consisted of the metadata collection stage, two training trials, and 10 test trials. Training trials were used to test the orientation and loudness levels of headphones and acquaint participants with the experimental setup. The experimental interface employed psiTurk (Gureckis et al., 2016) back-end with a web-based interface designed using jsPsych toolbox (de Leeuw, 2015). In each test trial, the participants simultaneously listened to two scenes in the two ears played dichotically and moved a cursor on the screen toward the left or right to report which side they were focusing on in each instant. Participants were asked to keep the cursor in the center when they focused on both scenes or neither. Vertical lines separated the screen into three parts (left, right, and center) to inform the participants about the cursor position. Scenes were randomly paired without replacement for each participant, such that each of the 20 scenes was played once in 10 test trials. The pairing was randomized to present each scene with different opposing scenes across participants. The experiment lasted 30 min on average, excluding the optional breaks between test trials.

### 2.2 Behavioral data analysis

#### 2.2.1 Data quality control

As outlined in earlier work (Kothinti et al., 2021), a quality control process was performed on the collected data to remove participants with abnormal responses. The average switching rate, defined as the number of times a participant switches their attention from one side to another per second, was used as metric to flag and remove outlier data. Trials with average switching rates higher than the 90 percentile (2 switches/s) and lower than the 10 percentile (0.05 switches/s) of the overall switching rates were considered abnormal. Participants with more than five abnormal trials were considered outliers and removed from subsequent data analysis. Removing participants based on this cut-off resulted in 101 and 107 participants for the *fwd* and *bwd* experiments, respectively.

#### 2.2.2 Average behavioral salience

Responses from each trial represented attentional switches toward one of the scenes in the dichotic listening paradigm. Responses were assigned a value of 1 when participants listened to a given scene and 0 otherwise. The resulting behavioral salience for each scene was derived by averaging responses from all participants for that scene (and different opposing scenes). All individual responses were adjusted by 1 s to account for an average participant reaction time delay, based on estimates from earlier findings (Huang and Elhilali, 2017). Mapped responses were then averaged across participants, followed by three moving window average operations of duration 1.5 s, to convert the responses to a temporal map in the [0, 1] range. This smoothed temporal map is called *the average behavioral salience* and reflects the attentional map for the scene over time. Figure 1B shows an example of individual participant responses and averaged behavioral salience for that scene across subjects. For the *bwd* experiment, the behavioral response for each scene was time-reversed (back to a forward time axis) for all subsequent analyses.

#### 2.2.3 Salient events

Salient event onsets and offsets, extracted from the average behavioral salience, were moments where attentional switches happen toward or away from a scene. To extract onsets and offsets, a derivative of the average behavioral salience was computed using first-order difference. Salient event onsets were derived as the locations of the local maxima of this derivative. For each onset, the offset was defined as the immediate local minimum of the derivative following the maximum. The slope of the derivative at the onset represents the fraction of participants responding to the event. Absolute consensus computed as the maximum of the average behavioral salience between onset and offset reflects the fraction of subjects responding to the event. A real-valued strength for each event was defined as the sum of the slope of the derivative at the onset and the absolute consensus scaled by 75 percentile of the overall onset slopes from all the stimuli. The event duration was computed as the difference between the offset and onset timestamps.

#### 2.2.4 Individual reaction times

Individual reaction times for each participant were measured with respect to salient event onsets (defined above). For each participant, the individual reaction time was defined as the time elapsed from the event onset to when the participant switched their attention toward the scene. If the participant did not change their attention 1 s before or after the event onset, they were considered non-responsive to the event. The choice of 1s before the onset reflected the reaction time adjustment. Other reasonable choices around 1 s resulted in quantitatively and qualitatively similar results. For each participant, reaction times were averaged across responsive events resulting in individual reaction times per participant.

#### 2.2.5 Forward/backward agreement

The agreement between continuous responses from *fwd* and *bwd* experiments was analyzed using a rank-based correlation metric. For each scene, *fwd* salience responses were correlated with the corresponding time-reversed *bwd* salience response, using Spearman correlation. This correlation served as a measure of agreement between responses from playing a scene in forward and backward directions.

In addition to continuous salience curves, agreement in timing between *fwd* and *bwd* discrete salient events was also analyzed. *bwd* event boundaries were reversed in time by subtracting the onsets and offsets from the scene duration to match with *fwd* time stamps as shown in Figure 1D. For each *fwd* event, the maximum overlap with *bwd* event was computed by comparing with all *bwd* events for the corresponding scene. An overlap score for each *fwd* event was calculated by normalizing the maximum overlap with the event duration. *fwd* events with more than 50% normalized overlap were called *forward-matched* events (referred to hereafter as *fwd-match* ), and the remaining events were called *forward-mismatched* events (referred to hereafter as *fwd-mismatch* ) as shown in Figure 1D. A similar analysis was carried out for *bwd* events, resulting in *bwd-match* and *bwd-mismatch* events. While the 50% threshold was chosen heuristically, similar results were obtained for different reasonable thresholds around 50%.

### 2.3 Acoustic and semantic analysis

#### 2.3.1 Acoustic analysis

Sixteen acoustic cues were used to analyze the effect of low-level acoustic dimensions on salience, which are outlined in Table 2. While these features represent a set of handcrafted attributes of the signal, they cover an exhaustive range of characteristics than span spectral, temporal, and spectro-temporal characteristics of the audio. Loudness was measured using bark-scale-based filter banks captured energy over time (Zwicker et al., 1991). Pitch and harmonicity quantified the harmonic nature of sounds as a function of time and were measured using a template matching algorithm (Goldstein, 1973). Spectral centroid and bandwidth captured first and second-order statistics, respectively, of the spectral profile of the acoustic signal. Spectral irregularity and flatness measured the smoothness of the spectral shape. Several statistics of spectro-temporal modulations were computed using a spectro-temporal cortical model (Wang and Shamma, 1994). These metrics included centroids and maximum energies for scale (or spectral modulations) and rate (or temporal modulations). High (>20 Hz) and low (<20 Hz) rate modulations were computed by averaging energies within the respective bands. The features were first computed at 8 ms sampling period and were later downsampled to 64 ms sampling period by taking the average of eight non-overlapping frames and *z*-score normalized per scene. Changes in these features were analyzed around salient event onsets to examine the relation between acoustic changes in the scene and attentional switches. Changes in features were defined as the difference between the average feature in the interval 0.5–1.0 s before and after the event. Feature changes were analyzed for matched and mismatched events from the *fwd* and *bwd* experiments to explore differences between these two classes of events.

**Table 2:** Description of features used in the acoustic analysis.

**Feature**	**Acronym**	**Description**
Loudness	LD	Average energy in bark-based decomposition
Spectral energy	SE	Energy in the spectrogram bands
Rate-scale energy	RSE	Energy in rate-scale (i.e., spectrotemporal modulation) decomposition
Brightness	BR	Spectral centroid
Bandwidth	BW	spectral spread around spectral centroid
Flatness	FL	Ratio of the geometric mean to the arithmetic mean of spectral magnitudes
Irregularity	IR	Measures jaggedness in the spectrum
Pitch	P	Pitch value based on template matching
Harmonicity	H	Degree of harmonicity as a measure of the strength of voicing
Maximum scale energy	MS	Maximum energy across all scales
Centroid of scales	CS	Scale centroid computed from scale decomposition
Maximum rate energy	MR	Maximum energy across all rates
High rate energy	HR	Measures roughness as energy in rates >20 Hz
Low rate energy	LR	Energy in rates ≤ 20 Hz
Centroid of rates	CR	Rate centroid computed from rate decomposition
Centroid with absolute rates	CAR	Rate centroid computed with the magnitude of rate in the weighted average

#### 2.3.2 Semantic analysis

For semantic analysis of the scenes, the open-source EfficientAT audio tagging model (Schmid et al., 2023) was used. EfficientAT^1^ is a deep convolutional neural network (CNN) based on the MobileNetV3 architecture (Howard et al., 2019), trained on 2 million human-labeled 10-s audio segments to identify the presence of 521 audio classes from the AudioSet ontology (Gemmeke et al., 2017). The model used a time-frequency representation of the audio segments as input and produced a posterior probability over the audio classes. The model architecture consisted of 17 CNN layers with inverted residual blocks (Sandler et al., 2018) and a fully connected output layer.

EfficientAT was used to extract semantic features by considering different semantic abstractions as represented by different layers of the model, with the final layer corresponding to class-level information. The final global average pooling layer was removed to preserve the temporal resolution. During the forward pass through the network, for each layer, *l*∈[1, 18], the hidden vector denoted by $y_{l}\in R^{T\text{x}K_{l}}$ was computed, where *K*_*l*_ was the flattened dimension of the *l*^*th*^ layer and *T* denoted the time samples. Since outputs of intermediate layers tend to be extremely large (>10^5^ in some layers), a layer-wise surprisal was computed for each layer to compress the information to 1-dimension and capture differences between the present and immediate past representations (Huang et al., 2018). Surprisal for layer *l*, at time *t*, was defined as the Euclidean distance between output *y*_*l, t*_ and the average output over the past 4 s. The 4 s past context was chosen based on performance on the salience prediction performance, and other choices of the past context around 4 s yielded similar quantitative results.

EfficientAT was also used to determine semantic labels for arbitrary segments of a scene. A segment under consideration was assigned a dominant class label from the five top-level root nodes in the AudioSet ontology: Human (speech, vocalizations, etc.), Things (vehicles, tools, etc.), Music (musical instruments, pieces, etc.), Animals (animals, birds, etc.), and Background (noise, babble, etc.). Source-ambiguous sounds (ex: impact sounds) and Channel, environment, and background sounds in the ontology were merged into the Background category. For finding the dominant class, the top 10 classes with maximum average posterior probabilities within the segment were considered candidate classes. Posterior probabilities of these classes were added to the top-level root node to propagate the beliefs. The top-level class with maximum probability after the belief propagation was considered the dominant class within the segment. This labeling method was carried out for the segments from *fwd* scenes.

In order to ensure that the backwards scenes were not disadvantageously processed using the semantic neural network model, we evaluated the model separately on the *fwd* and *bwd* scenes. The evaluation extracted the dominant 10 classes for each scene presented in *fwd* and *bwd* fashion based on posterior values; then, compared the predictions of *bwd* scenes with *fwd* and identified hits and misses. A recall score of 80% was noted across all scenes.

#### 2.3.3 Regression models

Regression models predicting average behavioral salience were developed using acoustic and semantic features. All three representations (behavioral salience, acoustic features, and semantic surprisals) were resampled at a sampling period of 64 ms. The resampled features were smoothed with moving-average smoothing, with different averaging lengths for acoustic and semantic features, which were treated as hyperparameters.

The regression model predicted each point *r*_*t*_ on the salience curve as a linear mapping from acoustic features **a**_*t*_ and semantic features **s**_*t*_. The features were sampled to include past context and future context to incorporate long-term context and were flattened into vectors of dimension *d*_*a*_ for acoustic features and *d*_*s*_ for semantic features (see **Figure 3A**). The lengths of past and future contexts were considered hyperparameters. The model can be mathematically written as


$$\begin{array}{l} \;{\hat{r}}_{t}=W_{a}^{T}a_{t}+W_{s}^{T}s_{t}+b\\ \;W_{a},a_{t}\in R^{d_{a}},W_{s},s_{t}\in R^{d_{s}}\\ W_{a},W_{s},b=\underset{W_{a},W_{s},b}{\arg\min}\sum_{i=1}^{N}\sum_{t=0}^{T_{i}}||r_{i,t}-{\hat{r}}_{i,t}|{|}_{2}^{2}+\lambda_{a}||W_{a}|{|}_{2}^{2}+\lambda_{s}||W_{s}|{|}_{2}^{2} \end{array}$$

**W**_*a*_, **W**_*c*_and*b* were estimated to minimize the Euclidean distance between behavioral salience *r*_*t*_ and predicted salience ${\hat{r}}_{t}$. Ridge regression with L_2_-regularization on the parameters was utilized for parameter estimation to avoid model overfitting (Hoerl and Kennard, 1970). Pearson correlation between behavioral salience and predicted salience evaluated model performance.

Three linear regression models with different input features were trained and evaluated. The first model, referred to as the acoustic-only model, used only acoustic attributes with a limited temporal context of up to 0.5 s. The second model, referred to as the acoustic-context model, used a longer context of up to 4 s for the acoustic features. The third model, referred to as the acoustic-semantic model, used acoustic and semantic features context.

Regression models were evaluated on 8 s segments of the stimulus/behavioral response with 4 s overlap (ignoring the first and last 1 s to avoid edge effects), resulting in 547 segments for both *fwd* and *bwd* data. If a matched (or mismatched) event overlapped with a segment, the segment was labeled matched (or mismatched). When a segment overlapped with both matched and mismatched events, the category with higher overlap was assigned to the segment. Segments with no overlap with any events were placed in a non-event category. Assigning event categories to *fwd* segments gave 59% *fwd-match* , 34% *fwd-mismatch* , and 7% no-event segments. Similarly, *bwd* segments were split into 60% *bwd-match* , 33% *bwd-mismatch* , and 7% no-event segments.

The regression model parameters were estimated using the 55 scenes from the DNSS-Ext dataset (Kothinti et al., 2021). DNSS-Ext dataset was collected using the same paradigm used in the present study. The DNSS-Ext dataset was split into 10-folds, and models were trained by leaving 1-fold out. Hyper-parameters such as the smoothing on the features, the temporal context length, λ_*a*_, andλ_*c*_ were chosen based on the correlation on the left-out fold. Once the hyper-parameters were fixed, the model parameters were estimated using the data from 55 scenes. We also evaluated the model using a different approach. Two sets of regression models for *fwd* and *bwd* data were trained using a 10-fold validation. By training on nine folds and testing on the left out fold, predictions were computed for the *fwd* and *bwd* data with their respective models. This second evaluation method yielded statistically similar effects as the first evaluation using the independent dataset.

#### 2.3.4 Salient event detection

In addition to predicting continuous salience, the linear filter framework was used to train a model to predict the *timing* of salient event onsets (i.e., a detection framework). In this analysis, the goal is not to produce a continuous estimate of salience as the scene unfolds but to derive binary outcomes about the onset timing of sound events. This approach is consistent with other works from the literature and allows us to directly gauge how the proposed acoustic and semantic features compare to other conceptual accounts of salience and sound event detection. We adopted the regression pipeline above with acoustic and/or semantic features to detect the events. Given the changed scope and limited data relative to degrees of freedom for the detection model, the event detection model differed from the regression model in a few ways: (i) a first-difference was applied to the features as the first step, (ii) the temporal weights were shared across the acoustic/semantic features, (iii) a sigmoid non-linearity was applied as the output function to convert the detection signal to a Bernoulli variable in the range ∈(0, 1). The detection model predicted if an event onset occurred within an input segment of 1 s. Two variations of this model were considered for evaluation. The first model, referred to as the acoustic model, used only the acoustic features within the 1 s duration and was referred to as the Acoustic model. The second model (A-S model) utilized acoustic and semantic features within the segment with a past context of 3s appended to the 1 s segment. For both these models, a stochastic gradient descent algorithm using a cross-entropy loss function on the 55 scenes from the DNSS-Ext dataset estimated model parameters. The model was trained using a mini-batch size of 200 segments, with a learning rate of 0.01 for 100 epochs through the whole training data. The model with the lowest training loss was used as the final model.

The performance of the A-S and A-only models was compared with other models from the literature. EfficientAT is primarily an audio tagging system and provides outputs that reflect changes in sound events. EfficientAT posteriors were thresholded at 0.5 and were smoothed using a median filter of width 1 s to provide smoothed binary labels indicating the presence of the audio classes. A first-order difference followed by summing across the classes resulted in a detection signal indicating changes in the class distribution. A second comparison was performed using the salient event detection model from Kim et al. (2014). The model was implemented using Bark filterbank features combined with linear discriminant weights trained on the DNSS-Ext dataset. Additionally, the salience map from Kayser et al. (2005) was used to generate a detection signal by summing across the frequency dimensions of the salience map and taking a first-order derivative. Finally, an interobserver agreement, computed by finding the number of subjects who moved their attention toward the scene within each segment, served as the detection signal with a theoretical upper bound on achievable performance.

Receiver operating characteristic (ROC) and area under ROC (AUROC) were used for evaluating performance. A range of thresholds was applied to the detection signals from each model to produce onset estimates. By comparing the estimates with the reference, the fraction of hits and false alarms (FA) were computed for each threshold.

## 3 Results

### 3.1 Behavioral analysis of forward and backward scenes

Overall, participants responded with a similar reaction time to both *fwd* and *bwd* scenes. Individual reaction times characterized the delay between a salient event and when each participant responded to that event. For the *fwd* experiment, the median participant reaction time was 0.91 s (std = 0.14 s), and for the *bwd* experiment, the median participant reaction time was 0.89 s (std = 0.15 s). The distributions of individual reaction times for *fwd* and *bwd* scenes were not significantly different (two-sided rank-sum test, *p* = 0.47).

The congruence of behavioral salience responses between *fwd* and corresponding *bwd* scenes was variable, with an average Spearman correlation ρ = 0.540 (std = 0.23) over 20 scenes. Randomly pairing *fwd* and *bwd* salience responses provided a noise floor for this correlation with an average ρ = 0.06 (std = 0.05) over 100 random permutations. Across the 20 scenes, correlations were widely variable (as low as ρ = 0.041 (*p* = 4e-10) for scene 10 and as high as ρ = 0.869 (*p* < 1e-100) for scene 20 (see **Table 5**). Figure 1C shows average behavioral saliences for scene 1 from *fwd* and *bwd* responses, yielding a correlation ρ = 0.587 (*p* < 1e-100). While there was no clear factor explaining this variability, the range of correlations suggested a strong interplay of scene acoustics, sparsity, context, and semantics, as will be explored in subsequent analyses.

Salience responses were also analyzed anchoring on events representing attentional switches toward (onset) and away from (offset) from the scene. On average, salient events in *fwd* and *bwd* scenes were comparable in number and duration of events. There were 451 events in *fwd* and 460 events in *bwd* responses, with an average of 23 events per scene and one event approximately every 5s. The average event duration of *fwd* events was 2.75 s (std =1.46 s) and 2.63 s (std = 1.28 s) for *bwd* events, with no statistically significant differences in duration between *fwd* and *bwd* events (two-sided rank-sum test, *p* = 0.20). The average strengths of *fwd* events (avg = 5.8e-4, std = 3.2e-4) and *bwd* events (avg = 5.5e-4, std = 3.0e-4) were also not statistically significant (two-sided rank-sum test, *p* = 0.07).

Looking closer at event types, we broke down events into matched and mismatched categories for both experiments (see Methods). We noted that the ratio of matched/mismatched events was similar for *fwd* and *bwd* scenes. For *fwd* events, there were 63% matched and 37% mismatched events, whereas, for *bwd* events, there were 61% matched events and 39% mismatched events. With that, divergent patterns emerged between matched and mismatched categories. The matched and mismatched categories were analyzed for differences in duration and strengths as shown in Figure 1E and summarized in Table 3. For both *fwd* and *bwd* events, matched events were shorter than mismatched events. Salience strengths for matched events were higher than mismatched events for both *fwd* and *bwd* events. In addition, slopes of matched events were higher than mismatched events, indicating lower temporal agreement for mismatched events compared to matched events. The absolute consensus for matched events was not significantly different from mismatched events, indicating that a similar percentage of participants reacted to matched and mismatched events. Thus, mismatched events showed variability when participants responded to an event onset, but on average captured the attention of the same number of participants as matched events.

**Table 3:** Event attributes for *fwd* and *bwd* events divided by event type.

	***fwd*-*match* (avg)**	***fwd*-*mismatch* (avg)**	***p***	***bwd*-*match* (avg)**	***bwd*-*mismatch* (avg)**	***p***
Duration (s)	2.51	3.17	**0.02**	2.46	2.90	0.16
Strengths	6.2e-4	5.2e-4	**4e-3**	6.0e-4	4.5e-4	**6e-5**
Slopes	3.9e-4	2.9e-4	**2e-3**	3.8e-4	2.4e-4	**1e-7**
Consensus	0.47	0.46	0.20	0.45	0.44	0.41

The *p*-values are computed using two-sided rank-sum tests. Statistically significant differences are shown in bold.

### 3.2 Acoustic dimensions driving behavioral salience for forward and backward events

**Figure 2:**
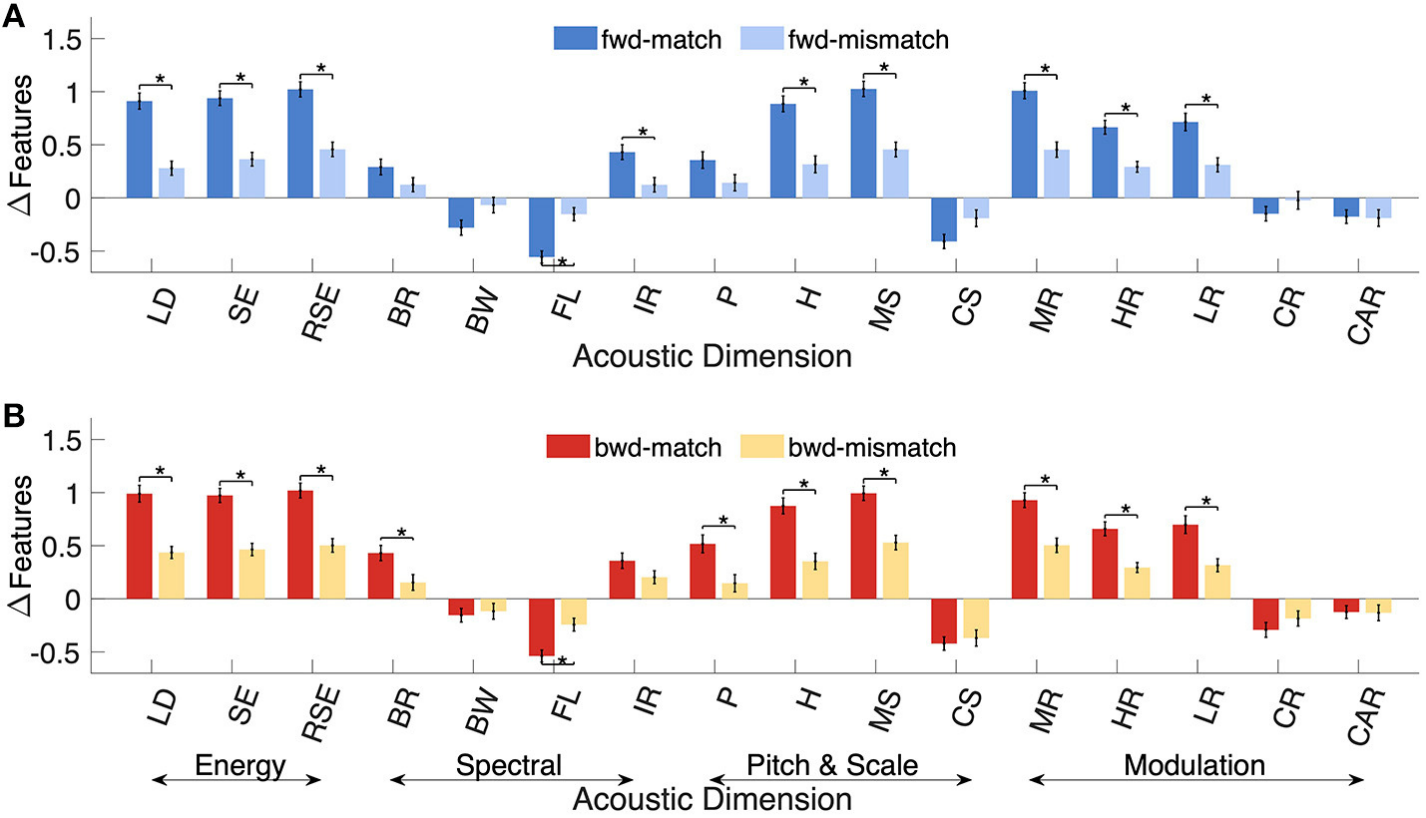
Feature changes around event onsets for **(A)** *fwd-match* and *fwd-mismatch* events. **(B)** *bwd-match* and *bwd-mismatch* events. Acoustic features are described in Table 2. The grouping of features based on their central characteristic is represented using arrows. The error bars represent ± 1 standard error. Statistically significant differences between the two groups are indicated with * after *post-hoc* HSD correction.

Changes in the acoustic profile of the scene were likely a factor in driving switches in attentional focus when listening to natural scenes. Changes in acoustic features before and after a salient event were quantified and contrasted for matched and mismatched events for *fwd* scenes as well as *bwd* scenes. We first performed an ANOVA to evaluate the differences in acoustic feature changes when grouped by the effects of scene presentation (*fwd* /*bwd* ), event type (matched/mismatched), and feature type. The ANOVA reveals that event type [*F*_(1, 14527)_ = 183.5, *p* = 1e-41], feature type [*F*_(15, 14527)_ = 123.24, *p* < 1e-100] groups showed significant effects and presentation type [*F*_(1, 14527)_ = 0.44, *p* = 0.50] did not show significant effects. The interactions among event and feature types significantly affected the acoustic changes [*F*_(15, 14527)_ = 16.67, *p* = 3e-44]. Interactions between presentation and event types [*F*_(1, 14527)_ = 0.02, *p* = 0.90] and presentation and feature types [*F*_(15, 14527)_ = 0.89, *p* = 0.58] were not significant. Next, we evaluated changes in individual acoustic features near event onsets, as shown in Figure 2. Table 4 summarizes the statistics quantifying whether the change in that feature is significantly different than zero or not. Table 5 recaps statistics from individual ANOVA tests for each acoustic attribute, exploring the effects of scene presentation and event type. Table 6 presents comparisons across different groups with p-values corrected for multiple comparisons. The features examined in this study spanned five general categories; energy, spectral, pitch, spectral, and temporal modulations.

**Table 4:** Statistics for feature change analysis.

**Feature**	***fwd-match***		***fwd-mismatch***		***bwd-match***		***bwd-mismatch***	
	***t*(284)**	***p***	***t*(165)**	***p***	***t*(278)**	***p***	***t*(180)**	***p***
LD	12.1	**1e-27**	4.2	**2e-05**	12.6	**3e-29**	7.7	**5e-13**
SE	13.7	**2e-33**	5.7	**3e-08**	14.7	**1e-36**	8.0	**6e-14**
RSE	14.4	**4e-36**	6.7	**1e-10**	14.6	**1e-36**	7.9	**2e-13**
BR	4.0	**5e-05**	1.9	**0.03**	6.0	**3e-09**	2.1	**0.02**
BW	−4.0	**4e-05**	−0.9	0.18	−2.4	**8e-03**	−1.6	0.06
FL	−9.7	**1e-19**	−2.5	**7e-03**	−9.6	**3e-19**	−4.0	**4e-05**
IR	6.2	**1e-09**	1.8	**0.04**	5.0	**6e-07**	3.3	**5e-04**
P	4.6	**4e-06**	1.9	**0.03**	6.2	**1e-09**	1.8	**0.04**
H	11.9	**3e-27**	4.0	**5e-05**	11.7	**3e-26**	4.6	**4e-06**
MS	14.3	**1e-35**	6.6	**2e-10**	14.7	**1e-36**	7.8	**3e-13**
CS	−6.2	**1e-09**	−2.4	**8e-03**	−6.7	**5e-11**	−4.9	**1e-06**
MR	13.5	**2e-32**	6.3	**1e-09**	13.4	**3e-32**	7.4	**2e-12**
HR	10.3	**1e-21**	5.8	**2e-08**	10.1	**5e-21**	6.3	**1e-09**
LR	8.7	**1e-16**	4.7	**3e-06**	8.4	**1e-15**	5.2	**3e-07**
CR	−2.2	**0.01**	−0.3	0.39	−4.2	**2e-05**	−2.6	**5e-03**
CAR	−2.8	**3e-03**	−2.4	**8e-03**	−2.1	**0.02**	−1.8	**0.04**

For each feature, the corresponding row shows statistics for a one-sided one-sample t-test. Statistically significant differences are shown in bold.

**Table 5:** ANOVA statistics for feature change analysis.

**Feature**	***fwd vs. bwd***		***Match vs. Mismatch***		**interactions**	
	**F(1,907)**	***p***	**F(1,907)**	***p***	**F(1,907)**	***p***
LD	2.3	0.13	58.5	**5e-14**	0.3	0.61
SE	0.9	0.33	60.4	**2e-14**	0.2	0.64
RSE	0.1	0.77	54.7	**3e-13**	0.1	0.74
BR	1.2	0.27	8.4	**4e-03**	0.5	0.47
BW	0.3	0.60	2.9	0.09	1.4	0.23
FL	0.3	0.56	32.2	**2e-08**	0.8	0.38
IR	0.0	0.96	9.9	**2e-03**	1.1	0.30
P	0.9	0.33	11.8	**6e-04**	0.9	0.35
H	0.0	0.87	46.7	**2e-11**	0.1	0.77
MS	0.1	0.78	49.4	**4e-12**	0.5	0.47
CS	1.7	0.19	3.5	0.06	1.3	0.25
MR	0.0	0.84	41.8	**2e-10**	0.7	0.39
HR	0.0	0.98	32.7	**1e-08**	0.0	0.95
LR	0.0	0.94	22.7	**2e-06**	0.0	0.90
CR	4.1	**0.04**	2.4	0.12	0.0	0.90
CAR	0.6	0.43	0.0	0.88	0.0	0.96

For each feature, the corresponding row shows statistics of ANOVA tests. Statistically significant differences are shown in bold.

**Table 6:** Statistics for feature change analysis with pair-wise comparisons.

**Feature**	***fwd-match vs. fwd-mismatch***		***bwd-match vs. bwd-mismatch***		***fwd-match vs. bwd-match***	
	***t*(449)**	***p***	***t*(458)**	***p***	***t*(562)**	***p***
LD	5.7	**7e-08**	5.1	**2e-06**	−0.7	0.85
SE	5.6	**5e-08**	5.4	**1e-06**	−0.4	0.98
RSE	5.3	**4e-07**	5.1	**3e-06**	0.0	1.00
BR	1.5	0.42	2.6	**0.05**	−1.4	0.45
BW	−2.0	0.17	−0.4	0.98	−1.3	0.50
FL	−4.5	**3e-05**	−3.5	**3e-03**	−0.2	1.00
IR	2.9	**0.02**	1.5	0.43	0.7	0.85
P	1.8	0.30	3.0	**1e-02**	−1.4	0.41
H	5.0	**4e-06**	4.7	**2e-05**	0.1	1.00
MS	5.3	**4e-07**	4.6	**4e-05**	0.3	0.98
CS	−2.1	0.15	−0.5	0.96	0.1	1.00
MR	4.9	**2e-06**	4.2	**4e-04**	0.8	0.83
HR	4.0	**3e-04**	4.1	**3e-04**	0.1	1.00
LR	3.4	**3e-03**	3.3	**5e-03**	0.1	1.00
CR	−1.2	0.65	−1.0	0.74	1.5	0.41
CAR	0.1	1.00	0.1	1.00	−0.6	0.93

For each feature, the corresponding row shows statistics of two-sample *t*-tests. Statistically significant differences are shown in bold. *p*-values were corrected for multiple comparisons using HSD correction.

Energy features analyzed the signal strength (or intensity) captured across different levels, including loudness (LD), spectral energy (SE), and spectrotemporal energy (RSE). These features were found to have significant changes at event onsets for *fwd-match, fwd-mismatch, bwd-match*, and *bwd-mismatch* events, indicating that subjects reacted to sudden increases in sound energy. When compared across groups, for all energy features, *fwd-match* and *bwd-match* events had a significantly higher magnitude of changes compared to *fwd-mismatch* and *bwd-mismatch* events, respectively. This indicated that changes around mismatched events were more subtle in intensity when compared to matched events. No significant differences were observed between *fwd-match* and *bwd-match* events for energy features.

Spectral features captured the timbral profiles of the scenes and included brightness (BR), bandwidth (BW), flatness (FL), and irregularity (IR). All four features considered showed significant changes for *fwd-match* and *bwd-match* events, with brightness and irregularity increasing at event onsets and bandwidth and flatness decreasing around events. For *fwd-mismatch* and *bwd-mismatch* events, while changes in BR, FL, and IR were significant, BW did not have significant changes. *fwd-match* events had a higher magnitude of changes compared to *fwd-mismatch* events only in FL, IR. In contrast, *bwd-match* events had a higher magnitude of changes compared to *bwd-mismatch* only for BR and FL features. No significant differences were observed between *fwd-match* and *bwd-match* events for the spectral features.

Pitch (P) and harmonicity (H) features captured harmonicity and voicing information in the scenes. Both features increased near event onsets for all event groups. While, *fwd-match* and *bwd-match* events had significantly higher changes compared to *fwd-mismatch* and *bwd-mismatch* events for H, only *bwd-match* showed significant differences from *bwd-mismatch* for P. No significant differences were observed between *fwd-match* and *bwd-match* events for pitch and harmonicity.

Spectral modulation features captured changes in spectral variations in the scenes. Max-scale energy (MS) increased at event onsets, and scale centroid (CS) decreased around event onsets. *fwd-match* and *bwd-match* events had significantly larger changes compared to *fwd-mismatch* and *bwd-mismatch* events only for MS. No significant differences were observed between *fwd-match* and *bwd-match* events for the scale-related features.

Temporal modulations were captured using features reflecting average and maximum changes in temporal rates or variations in the scenes. Maximum rate energy (MR), high-rate energy (HR), and low-rate energy (LR) increased at event onsets. Rate centroid (CR) had significant negative changes for *fwd-match* , *bwd-match* , and *bwd-mismatch* events and no significant changes for *fwd-mismatch* events. Absolute rate centroid (CAR), which ignored the direction of modulations, had significant negative changes for all categories of events. As with other energy features, MR, HR, and LR features showed significant differences between *fwd-mismatch* and *fwd-match* events and between *bwd-mismatch* and *bwd-match* events. Both CR and CAR showed no significant differences between *fwd-match* and *fwd-mismatch* as well as *bwd-match* and *bwd-mismatch* groups. No significant differences were observed between *fwd-match* and *bwd-match* events for the temporal features.

Overall, pairwise comparisons showed that mismatched events had lower acoustic changes compared to matched events for both *fwd* and *bwd* events. There were no significant differences between *fwd-match* and *bwd-match* events.

### 3.3 Accounts of auditory salience spanning acoustics, context, and semantics

**Figure 3:**
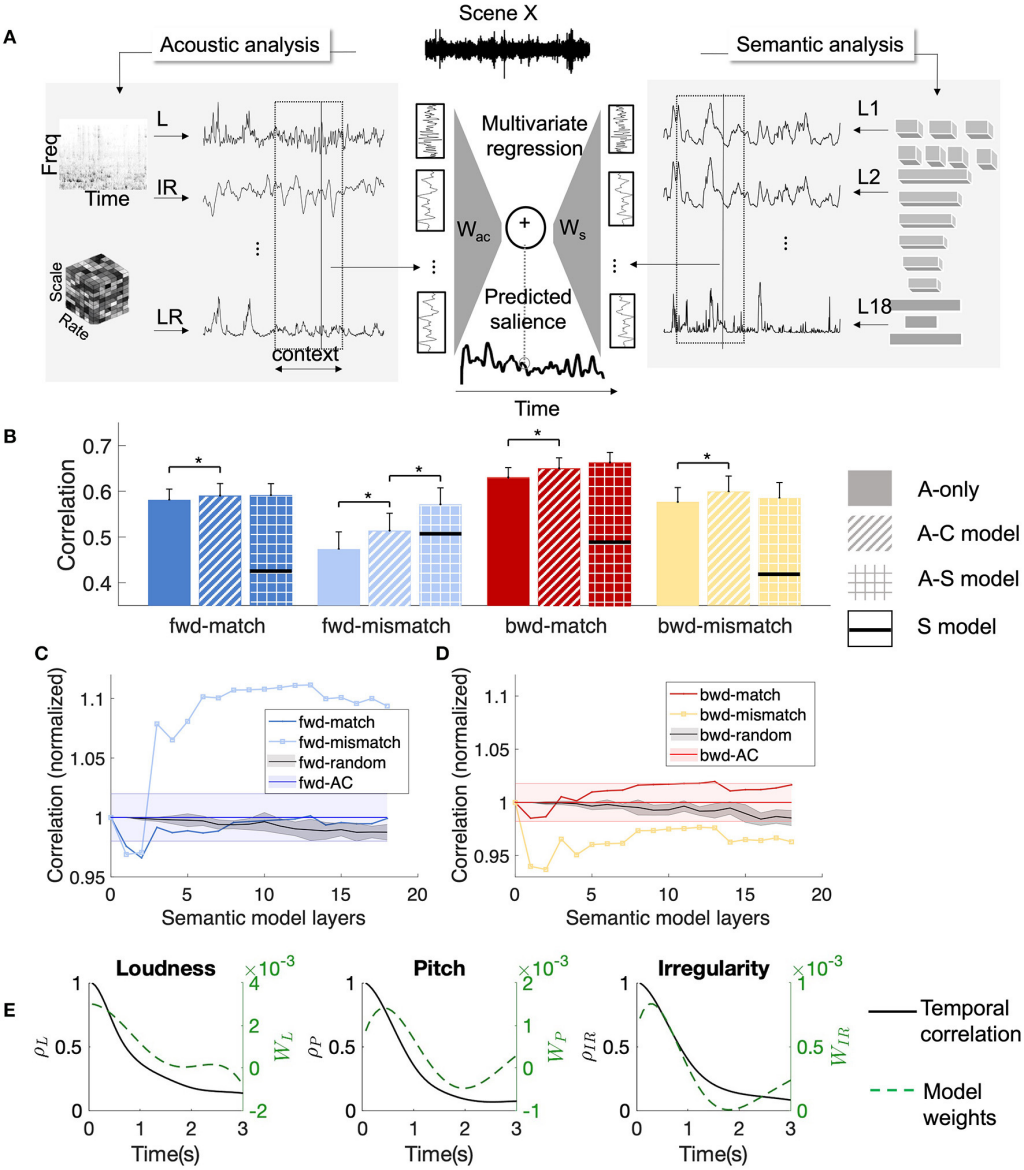
Regression model and performance. **(A)** Schematic of the model. A window of acoustic features and layer-wise surprisals was converted to a vector and was used as variables to predict the behavioral surprisal. **(B)** Correlations for *fwd-match* , *fwd-mismatch* , *bwd-match* , and *bwd-mismatch* segments. Statistically significant differences are indicated by *. The S-model performance is shown as a line in black. **(C)** Layer-wise model performance measured relative to A-C model for *fwd-match* and *fwd-mismatch* segments. Shaded in black is the model performance with random features added to A-C model, with the shaded area representing standard error across 10 random trials. Shaded in blue is the A-C model performance. **(D)** Layer-wise model performance measured relative to A-C model for *bwd-match* and *bwd-mismatch* segments. Shaded in black is the model performance with random features added to A-C model, with the shaded area representing standard error across 10 random trials. Shaded in red is the A-C model performance. **(E)** Autocorrelation function of the features (left *y*-axis) and the model weights (right *y*-axis) for three different features.

The feature change analysis was limited in showing driving factors of salience as several features presented were highly correlated (Kothinti et al., 2021). Regression models account for these correlations and provide more direct evidence for factors contributing to salience. Among the three models considered, the acoustic-only model (A-only model, refer to Figure 3B) showed the worst performance in general for all the event categories. For the acoustic-only model, the average correlation for *fwd* (avg = 0.557) was significantly lower (one-sided rank-sum test, *zval* = −2.04, *p* = 0.02) than *bwd* segments (avg = 0.618). When the segments were broken down by the matched-mismatched categories, performance for *fwd-match* segments (avg = 0.581) segments was significantly higher than *fwd-mismatch* segments (avg = 0.473) (one-sided rank-sum test, *zval* = 2.22, *p* = 0.01). Similarly, performance on *bwd-match* segments (avg = 0.631) was significantly higher than *bwd-mismatch* segments(avg = 0.577) (one-sided rank-sum test, *zval* = 1.99, *p* = 0.024). No significant differences were observed between *fwd-match* and *bwd-match* segment correlations (two-sided rank-sum test, *zval* = 1.62, *p* = 0.11). Thus, instantaneous acoustic features predicted behavioral salience better around matched events when compared to mismatched events for both *fwd* and *bwd* scenes.

Adding long-term context to acoustic features (A-C model, refer to Figure 3B) improved salience prediction. Segment correlations were improved for both *fwd* (avg = 0.581) and *bwd* (avg = 0.642) segments. Pairwise sign-rank tests indicated the improvements were significant for both *fwd* (one-sided sign rank test, *zval* = 5.15, *p* = 1e-7) and *bwd* (*zval* = 4.81, *p* = 1e-6) segments. Comparison between *fwd* and *bwd* segments indicated a significant difference in correlations (one-sided rank-sum test, *zval* = -1.99, *p* = 0.023). Breaking down by the events, adding long-term context significantly improved performance for *fwd-match* (avg = 0.591, sign-rank test, *zval* = 2.69, *p* = 0.003), *fwd-mismatch* (avg = 0.514, sign-rank test, *zval* = 3.43, *p* = 3e-4), *bwd-match* (avg = 0.650, sign-rank test, *zval* = 3.57, *p* = 2e-4) *bwd-mismatch* (avg = 0.599, sign-rank test, *zval* = 2.21, *p* = 0.01) segments. Despite the improvements in salience prediction with the long-term context, the differences between *fwd-match* and *fwd-mismatch* segments (one-sided rank-sum test, *zval* = 1.85, *p* = 0.03) and between *bwd-match* and *bwd-mismatch* segments (one-sided rank-sum test, *zval* = 1.96, *p* = 0.025) remained statistically significant. Significant differences were observed between *fwd-match* and *bwd-match* segment correlations (one-sided rank-sum test, *zval* = 1.96, *p* = 0.037). The relative improvements in average correlations from using the long-term context were higher for *fwd-mismatch* (9%) in comparison to *fwd-match* (2%), *bwd-match* (3%), and *bwd-mismatch* (4%). Thus, acoustic features with long-term context predicted salience better than those without context across all the segments and categories. The effect was strongest for *fwd-mismatch* segments, as indicated by the improvement in correlation. The addition of long-term context reduced the differences between *fwd-match* and *fwd-mismatch* events.

The acoustic-semantic model (A-S model), which used semantic features in addition to acoustic features with long context, provided further gains in prediction. Adding semantic features improved prediction for *fwd* (avg = 0.600, sign-rank test, *zval* = 1.92, *p* = 0.03) and *bwd* segments (avg = 0.644, sign-rank test, *zval* = 0.02, *p* = 0.98), with only the improvements on *fwd* segments being significant. For this model, segment correlations for *fwd* were not significantly different from *bwd* (two-sided rank-sum test, *zval* = 1.44, *p* = 0.15). The further breakdown of *fwd* segment correlations revealed that the gains from the A-S model (compared to the A-C model) were significant only for *fwd-mismatch* segments (avg = 0.562, sign-rank test, *zval* = 3.33, *p* = 4e-4). For other event groups, the difference in correlations between the A-S and A-C models was not significant (sign-rank test: *fwd-match* (avg = 0.590, *zval* = 0.8, *p* = 0.38), *bwd-match* (avg = 0.661, *zval* = 0.22, *p* = 0.82), *bwd-mismatch* (avg = 0.577, *zval* = 1.11, *p* = 0.27)). With the contribution from the semantic features, segment correlations for *fwd-mismatch* segments were not statistically different from *fwd-match* correlations (rank-sum test, *zval* = 0.39, *p* = 0.69), while *bwd-mismatch* segments were predicted significantly worse than *bwd-match* segments (rank-sum test, *zval* = 2.34, *p* = 0.02). Thus, the semantic features were only effective in improving prediction for *fwd-mismatch* segments, supporting our hypothesis that the mismatched events from *fwd* scenes were strongly driven by semantic attributes of the scene.

To analyze the gains from the semantic surprisals, we tested regression models which incrementally included surprisals from different layers, from input layers to classification layers. Figures 3C, D compares the prediction performance with each layer cumulatively added to the lower layers. The performance gains added by incremental addition of higher layers were mostly prominent for *fwd-mismatch* segments. To account for the added parameters with the addition of more layers, we trained randomized baseline models by incrementally adding random values for surprisals. Performance of the randomized baseline models stayed constant for all the layers, which further cements the benefits of the layer-wise surprisals. When only the semantic surprisal was used for predicting the behavioral salience (S model), the performance across all segments was lower than the A-S model.

To further understand the gains from the long-term context, we analyzed temporal correlations for individual features, focusing on segments around events. In Figure 3E, the temporal correlations represent the normalized autocorrelation values of each feature as a function of the delay computed from all the scenes. The autocorrelation showed a slow decay in magnitude over the temporal lag. The temporal weights from the model for individual features, shown as temporal response functions, seemed to follow the autocorrelation function closely, thus tracking the dynamics of the features. Since the model was trained to mimic human response, we can infer that the human response also takes long-term feature dependencies into account.

### 3.4 Classwise analysis of forward *matched* and *mismatched* events

**Figure 4:**
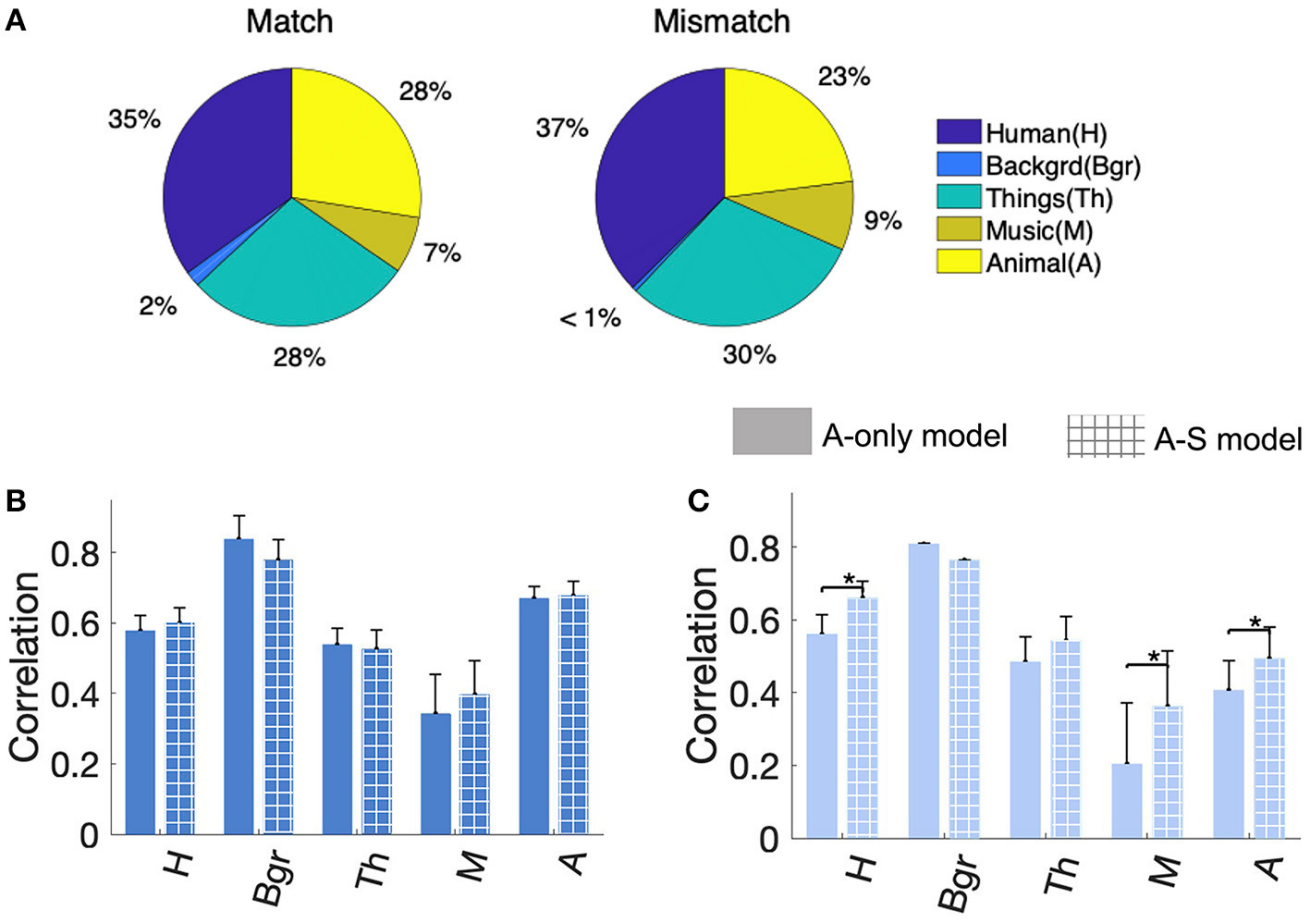
**(A)** Classwise breakdown of the *fwd-match* and *fwd-mismatch* segments. **(B)** Correlations of *fwd-match* segments for A-only and A-S models. **(C)** Correlations of *fwd-mismatch* segments for A-only and A-S models. Error bars indicated ±1 standard error in fraction estimate. Statistically significant differences are marked with a *.

Further characterization of the salience prediction examined the effects of sound classes. Examining the matched-mismatched segmentation and class labels revealed that mismatched segments had a higher percentage of Human, Things, and Music classes when compared to matched (Figure 4A). Figures 4B, C show the class-wise performance breakup for *fwd-match* and *fwd-mismatch* segments, respectively. The segment correlations were analyzed for A-only and A-S models. A wide range of correlations was observed across different classes, and the improvement from the semantic models was not uniform across classes. In *fwd-match* segments, there were no significant differences in performance between A-only and A-S models (one-sided sign-rank test, H: *zval* = 1.47, *p* = 0.14, Bgr: *zval* = 0.73, *p* = 0.46, Th: *zval* = 0.81, *p* = 0.41, M: *zval* = 0.24, *p* = 0.80, A: *zval* = 1.15, *p* = 0.25). In *fwd-mismatch* segments, Human, Music, and Animal classes showed significant improvements with the A-S model (one-sided sign-rank test, H: *zval* = 3.41, *p* = 3e-4, M: *zval* = 2.41, *p* = 0.008, A: *zval* = 2.22, *p* = 0.01) and the remaining classes showed no significant differences (Bgr: *zval* = 0, *p* = 0.99, Th: *zval* = 1.33, *p* = 0.09). These differences in class-wise performance indicated a higher-order dependence on semantics that is class-dependent.

### 3.5 Effect of context and semantics for event detection

**Figure 5:**
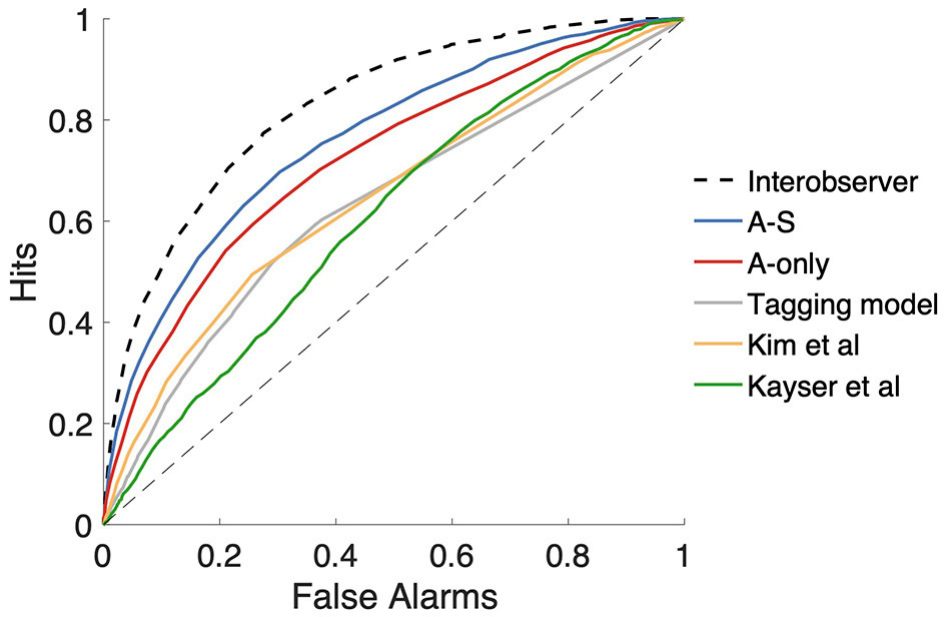
Salient event detection performance of different models on the forward data shown as ROC curves.

The event detection paradigm was used to quantify the benefits of context and semantics in predicting event onsets. Salient event detection using models with acoustic and semantic cues was compared with several existing models of salient event detection for this purpose. As seen from the ROC curves in Figure 5, long-term context and semantic features (A-S) provided the best detection performance on the *fwd* data with AUROC of 0.761. The A-S model showed a significant improvement over the acoustic (A-only) model (AUROC = 0.723), validating the benefits of semantics and acoustic context. In comparison, a detection model using loudness from bark filters (Kim et al., 2014) (AUROC = 0.646) performed poorly, indicating that low-level features considered in this study were better at predicting events than simple time-frequency representations. Similarly, the center-surround analysis from Kayser et al. performed worse (AUROC = 0.608), indicating that local contrast does not characterize the salient events completely. Additionally, event detection using an audio tagging model was also found to be inferior (AUROC = 0.632) to the A-S model. The interobserver agreement has an AUROC of 0.801, showing a significant gap from the A-S model.

## 4 Discussion

Exploring the contextual and semantic effects on auditory salience in natural audio is the central focus of this study. Working with a dichotic listening paradigm and using time-reversed audio to contrast behavioral responses, we explored an experimental method to capture the higher-order effects of auditory salience. Overall, the experimental findings show that the conspicuity of acoustic events is perceived differently depending on their acoustic and semantic characteristics. For a subset of events with strong acoustic variations, behavioral responses were generally similar when presented normally or time-reversed. These matched events were not unique to any sound class and could be best predicted using rich representations of their acoustic attributes with temporal context. No additional information could be gleaned from their semantic features supporting the hypothesis that sound events with sufficient acoustic variability relative to their surrounds will stand out from the acoustic scene. This result is in line with a contrast theory that has been posited to underlie salience in other sensory modalities across multiscale and multirate features (Itti and Koch, 2001; Nothdurft, 2005; Wang et al., 2014). In contrast, other sound events with more nuanced or muted changes in acoustic attributes -relative to their context- can also stand out as salient. These events induce a different behavioral response when heard naturally or time-reversed. These mismatched events are also not unique to any sound category, but do benefit from some knowledge of sound semantics in order to improve their fit in predicting behavioral measures of salience.

Distinguishing effects of acoustic, contextual and semantic attributes leveraged the use of time-reversal using the same scenes. This experimental setup provides a comparative framework to assess how temporal context and semantic factors play a role in determining salience. Existing experimental paradigms tested the effects of temporal context and semantics on auditory salience as part of expectation-violation in sequences of tokens (Parmentier, 2008; Marsh et al., 2009; Hughes, 2014; Macken, 2014). These studies explored the contextual and semantic factors mostly with controlled or synthetic stimuli where the notion of semantics often contrasted simple tones with meaningful word, rather than a broader analysis of the semantic content an an entire scene. The present study aims to quantitatively assess contextual and semantic effects on auditory salience in natural scenes to address this gap.

To establish the contextual and semantic effects, we first demonstrated that the *bwd* experiment shows differences in human responses compared to *fwd* data. Comparing average behavioral salience using a correlation-based analysis showed a significant difference in how subjects reacted to the same scenes played in different directions. If salience was driven only by instantaneous acoustics, the responses from *fwd* and *bwd* (after reversing) should have a high agreement, as the acoustics remain similar when scenes are reversed. The lack of agreement points to additional factors other than acoustics playing a role. At the same time, various behavioral metrics such as reaction times, number of events, event durations, and event strengths were not significantly different, indicating a similar participation profile across the two experiments. The high variability in the correlations (min = 0.04, max = 0.87) across the scenes, which had a variety of objects, hints at a complex interplay of scene context and semantics along with acoustics.

In addition to the salience curves, we analyzed the temporal alignment between *fwd* and *bwd* events with a threshold on the amount of overlap (chosen as 50%). This analysis provides mismatched events, which are moments in time where the *fwd* and *bwd* events did not have a corresponding shift in attention in the opposite direction. The proposed temporal alignment process is a novel approach that can theoretically separate contextually or semantically driven events from purely acoustically driven events. The similarity in absolute consensus for matched and mismatched events across *fwd* and *bwd* data points to a consistent attention switch within the event. This separation of events provided an anchor to explore the role of acoustics and semantics.

Acoustic characterization provides an indirect validation of the effects of semantics on salient events. Acoustic feature changes around event onsets were previously (Huang and Elhilali, 2017) employed to measure the change in stimuli properties that may have caused an attention switch. The feature change analysis revealed how *fwd-mismatch* and *bwd-mismatch* events were driven by lower acoustic changes when compared to *fwd-match* and *bwd-match* events, respectively. While this effect was most evident in energy features, several other spectral and temporal modulation features showed similar trends. Features such as pitch, brightness, and roughness were observed to affect salience directly (Arnal et al., 2019; Bouvier et al., 2023), and the lower changes in these features for mismatched events suggest contribution from other factors. Additionally, none of the acoustic features considered showed significant differences between *fwd-match* and *bwd-match* events. Thus, the matched events for *fwd* and *bwd* are driven by similar acoustic changes, which are distinctly different from the mismatched events. The feature change analysis does not provide a measure of contributions from individual features due to the cross-correlations between the features (Kothinti et al., 2021).

For semantic characterization, this study employed changes in in abstract embeddings of the scenes that were trained to identify sound events in the scene using a large deep scale neural network. Using a state-of-the-art model trained on an audio tagging task, the network embeddings (or weights) reflect implicit features representations that generally reflect the semantic content of the acoustic scene and ultimately facilitate the tagging task for which the model is trained (LeCun et al., 2015). There is sparse prior work utilizing object or semantic information in auditory salience models. In the visual domain, early works used manual annotation of the object information (Einhauser et al., 2008) and object detection models (Cerf et al., 2009). Adopting these methods can be difficult owing to the ambiguity about the appropriate abstraction of objects and the time-intensive process involved in assigning such information in natural scenes. More recent works in visual salience used information from deep learning models to capture high-level information (Li and Yu, 2016). Along the same lines, recent developments in deep learning models for audio classification paved the way for utilizing large-scale models pretrained on general audio datasets such as AudioSet (Gemmeke et al., 2017). The multi-stage hierarchical nature of these models provides multiple levels of abstractions that closely correspond with various processing stages of the auditory cortex based on fMRI studies (Kell et al., 2018; Giordano et al., 2023). Based on similar assumptions, Huang et al. (2018) used surprisal from intermediate layers of an audio tagging model measuring semantic expectation-violation, which we adopted in this study. We reduced each layer to one dimension to accommodate the small training data at our disposal. Future exploration of computational accounts can build on our paradigm with other dimensionality reduction techniques, such as Kernel PCA as used by Giordano et al. (2023).

Prediction models such as linear regression take cross-correlations into account and provide a method to evaluate the relevance of individual features with the predicted variable. In this study, we used a linear model to predict salience from different features. By using a model with temporal weights and semantic features, we validated the effect of context and semantics in driving salience. This approach parallels several visual salience models that incorporated contextual and semantic factors using linear combinations of low-level and object-level salience (Cerf et al., 2009; Li and Yu, 2016).

The role of temporal context in salience prediction has been demonstrated in a number of studies. The present analysis shows that such context operates over larger time scales than originally reported in previous work. Earlier studies employed temporal context as a smoothing operation (Huang and Elhilali, 2017) over a few seconds or by using linear filtering on temporal context (Kim et al., 2014). These approaches were restrictive because of the simplicity of the smoothing or the amount of context used in the temporal filtering. Effectively, earlier models of contextual effects relied on a general averaging that operates across all low-level or high-level features under study. In the present study, the temporal context is learned through supervised training to infer relative weighting of individual acoustic or semantic attributes of the scene. By training the filters to predict salience from long-range stimulus properties, we aim to capture temporal processing used by humans in producing behavioral salience. The filter shape shown in Figure 3E highlights the filtering mechanism for different features that seems to span a few seconds. The autocorrelation of these features has a decay period of more than 3 s. The slow decay of the features when compared to the autocorrelation profiles is indicative of the way statistics were dynamically by humans.

While including long temporal context improved performance for different segment categories, as shown in Figure 3B, the degree of improvement was higher for *fwd-mismatch* segments than other segments. The context effect was stronger for *fwd-mismatch*, supporting our hypothesis that these segments were influenced by long-term temporal context. Secondly, adding semantic information offered a more striking result, with the improvement only observed for *fwd-mismatch* segments. While the acoustic feature change suggested both *fwd-mismatch* and *bwd-mismatch* events had lower acoustic changes, the semantic information only helped *fwd-mismatch* segments. These two observations provide strong evidence supporting our hypothesis that *fwd-mismatch* events were driven by high-level factors. Exploring the prediction performance for different event categories in the *fwd-mismatch* data indicated Human, Music, and Animal categories benefit from contextual and semantic information.

In this study, events from *bwd* data served as a counterpoint to *fwd* events. While *bwd-mismatch* events show lower acoustic changes than *bwd-match* , similar to *fwd-mismatch* events, they do not benefit from the semantic information. Although the results shown in Figure 3B are from models trained on 55 DNSS-Ext scenes played in the forward direction, qualitatively similar trends were observed even when models were trained with *bwd* data from this study. Thus, the *fwd-mismatch* events are distinct from *bwd-mismatch* events in that they are driven by semantics.

The event detection paradigm provides an alternative framework to evaluate salience models, where the focus is detecting the moments when subjects switched their attention. In addition, we used this framework to compare the salience models proposed in this study with some notable computational frameworks of salience. As noted previously by Huang and Elhilali (2017), the linear discriminant analysis on a wide range of acoustic features performs much better than using bark-scale-based time-frequency features and saliency maps inspired by visual salience models. The addition of semantics and long-term context improved the detection performance, similar to the salience prediction results. While the improvements from semantic features were explored in Huang and Elhilali (2018), the current study proposed a model with more flexibility in the amount of context used. An additional benefit of the event-based paradigm is the theoretical upper bound on the detection performance as indicated by the interobserver agreement. As seen from Figure 5, there is a significant gap between the interobserver agreement and the A-S model.

A natural question arising from the event detection framework is whether a model trained with a sound event detection objective is suitable for detecting salient events. A change detection analysis of the posteriors from the audio tagging model detected salient events better than chance but worse than the proposed model. The acoustics and intermediate representations from the tagging model are essential for salient event detection. Thus, the high-level information from the tagging model useful for salience constitutes more than the object categories. We note that the detection framework focuses only on the onsets as opposed to sound event detection paradigms, which consider event offsets as well (Mesaros et al., 2021). This difference resulted from the dichotic listening paradigm, which focuses on when the subjects shift their attention toward a scene and not away from a scene. The offsets derived from the behavioral data may not be precise moments when they moved away from the scene. But, if necessitated, the proposed framework can be extended to offset detection by expanding the output of the model to a 3-way classification for onset, offset, and “during event” probabilities.

The models proposed in this study for salience prediction and event detection use simple parametric formulations, which make them flexible to incorporate different types of semantic information with varied context lengths. While such simplicity can be limiting, it also enables easy incorporation of additional semantic abstractions. There is a possibility that more granular measures such as phonetic features and lexical surprisals could explain the salience of human sounds. The goal of building a comprehensive picture of salience has important technological consequences in addition to scientific benefits. Salience measures can be useful in associating a degree of perceptual confidence to audio events for applications such as audio event detection and audio captioning. More ready usage of salience in real-world applications requires further efforts to improve the computational models. The experimental data collected in this data can serve as a benchmark to evaluate and improve salience models such that they can closely match human experience in real-world scenarios.

## Data availability statement

The raw data supporting the conclusions of this article will be made available by the authors, without undue reservation.

## Ethics statement

The studies involving humans were approved by Johns Hopkins University Institutional Review Board. The studies were conducted in accordance with the local legislation and institutional requirements. The participants provided their written informed consent to participate in this study.

## Author contributions

SK: Conceptualization, Data curation, Formal analysis, Investigation, Methodology, Resources, Software, Validation, Visualization, Writing – original draft, Writing – review & editing. ME: Conceptualization, Data curation, Formal analysis, Funding acquisition, Investigation, Methodology, Project administration, Resources, Software, Supervision, Validation, Visualization, Writing – original draft, Writing – review & editing.

## Footnotes

^1^ https://github.com/fschmid56/EfficientAT
